# Alleviating effects of morin against experimentally-induced diabetic osteopenia

**DOI:** 10.1186/1758-5996-5-5

**Published:** 2013-02-06

**Authors:** Hatem M Abuohashish, Salim S Al-Rejaie, Khaled A Al-Hosaini, Mihir Y Parmar, Mohammed M Ahmed

**Affiliations:** 1 Department of Pharmacology and Toxicology, College of Pharmacy, King Saud University, P.O. Box 2457, Riyadh, 11451, Saudi Arabia; 2 Experimental Animal Care Center, College of Pharmacy, King Saud University, P.O. Box 2457, Riyadh, 11451, Saudi Arabia

**Keywords:** Diabetic osteopenia, Morin, Oxidative stress, Inflammation, Micro CT

## Abstract

**Background:**

Plant flavonoids are emerging as potent therapeutic drugs effective against a wide range of aging diseases particularly bone metabolic disorders. Morin (3,5,7,20,40-pentahydroxyflavone), a member of flavonols, is an important bioactive compound by interacting with nucleic acids, enzymes and protein. The present study was designed to investigate the putative beneficial effect of morin on diabetic osteopenia in rats.

**Methods:**

Streptozotocin (STZ)-induced diabetic model was used by considering 300 mg/dl fasting glucose level as diabetic. Morin (15 and 30 mg/kg) was treated for five consecutive weeks to diabetic rats. Serum levels of glucose, insulin, deoxypyridinoline cross links (DPD), osteocalcin (OC), bone specific alkaline phosphatase (BALP), telopeptides of collagen type I (CTX), interleukin 1 beta (IL-1β), interleukin 6 (IL-6), tumor necrosis factor alpha (TNF-α), thiobarbituric acid reactive substance (TBARS) and reduced glutathione (GSH) were estimated. Femoral bones were taken for micro CT scan to measure trabecular bone mineral density (BMD) and other morphometric parameters.

**Results:**

Significant bone loss was documented as the level of bone turnover parameters including DPD, OC, BALP and CTX were increased in serum of diabetic rats. Morin treatment significantly attenuated these elevated levels. Bone micro-CT scan of diabetic rats showed a significant impairment in trabecular bone microarchitecture, density and other morphometric parameters. These impairments were significantly ameliorated by morin administration. Serum levels of glucose, TBARS, IL-1β, IL-6 and TNF-α were significantly elevated, while the level of insulin and GSH was decreased in diabetic rats. These serum changes in diabetic rats were bring back to normal values after 5 weeks morin treatment.

**Conclusion:**

These findings revealed the protective effect of morin against diabetic induced osteopenia. We believed that this effect is through its both the anti-inflammatory and antioxidant properties.

## Background

Diabetes mellitus is a group of frequently encountered clinical metabolic problems. It is characterized by hyperglycemia due to shortage in the secretion and/or actions of insulin. Diabetic complications are many including nephropathy, neuropathy, retinopathy and cardiovascular diseases, as well as alterations of bone and mineral metabolism [1]. Diabetic associated osteopenia results in both increased risk of bone fracture and delay in the process of bone fractures healing, which affect the quality of life in diabetic patients [2,3]. Optimal therapies for such disorder are few. Therefore, controlling its development is of great significance for patients with diabetes.

Mechanisms responsible for diabetic osteopenia have not been clearly identified thus far. Oxidative stress has been suggested as one of the contributing factors in diabetic osteopenia [4]. Bai et al. [5] reported that, osteoblast or bone forming cells, differentiation can be inhibited by oxidative stress. It also believed that oxidative stress induces osteoblast insults and apoptosis [2]. Hyperglycemia itself showed damaging effect on osteoblast differentiation, bone formation and mineralization [6]. Furthermore, oxidative stress along with hyperglycemia are sources for the generation of free radicals and reactive oxygen species (ROS) that can disrupt cellular function and damage proteins, lipids and DNA [7]. Thus, the diabetic-induced oxidative stress control may significantly influence the incidence and development of diabetic osteopenia.

Morin is one of the naturally occurring bioflavonoids, originally isolated from members of the Moraceae family [8], mostly found in different herbs and fruits including onion, seed weeds, mill, fig, almond, red wine and Osage orange [9,10]. Morin exhibited several pharmacological properties such as antioxidant [11,12], anti-inflammatory [13], chemo-protective [14], anticancer [15], and anti-promotion [16]. Nandhakumar et al., [10] reported that, morin supplementation to cancerous rats significantly attenuated oxidative stress and brought the decreased of SOD, CAT, and GPx activities to normal levels. Also, morin has shown to protect various human cells, such as myocytes, endothelial cells, hepatocytes and erythrocytes, against free radical induced oxidative damages [17,18]. The chemical structure of morin and other bioflavonoids can be distinguished by the presence of two aromatic rings connected by c-pyrone ring where polar hydroxyl groups are bind at various positions. These hydroxyl groups are suggested to be responsible for the free radical scavenging properties shared by morin and other naturally occurring bioflavonoids [19]. Interestingly, another bioflavonoid, quercetin, was found to attenuate bone loss and osteopenia associated with experimentally induced diabetes in rodents [3]. One of the main gratitude of morin is for its very minimal toxicity even at higher dose usage [11,20]. Literature survey revealed that morin has not been tested for its osteoprotective affects. Because of its high abilities against several chronic and elderly diseases, this study is designed to investigate the possible alleviating effects of morin on diabetic induced osteopenia in male Wistar albino rats Scavenging.

## Materials and methods

### Animals

The present study was conducted in eight weeks old male Wister albino rats weighting 250–280 g were obtained from the Experimental Animal Care Center, College of Pharmacy, King Saud University, Riyadh, Saudi Arabia. They were housed under controlled environmental conditions (25°C and a 12 h light/dark cycle) and had free access to pulverized standard rat pellet diet (Manufactured by Grain Silos & Flour Mills Organization, Riyadh, Saudi Arabia) and tap water. All experimental procedures including euthanasia were conducted in accordance with the National Institute of Health Guide for the Care and Use of Laboratory Animals, Institute for Laboratory Animal Research (NIH Publications No. 80–23; 1996) as well as the Ethical Guidelines of the Experimental Animal Care Center, College of Pharmacy, King Saud University, Riyadh, Saudi Arabia.

### Diabetes induction

Diabetes was chemically induced by intraperitoneal (i.p.) injection of freshly prepared in 0.1 mol/L citrate buffered solution (pH 4.5) of streptozotocin (Sigma Aldrich, St. Louis, MO, USA) at a dose of 55 mg/kg body weight. Control (vehicle) rats were injected with equal volume of 0.1 mol/L citrate buffer. Four days after STZ injection, diabetes induction was confirmed by measuring fasting blood glucose level in a tail vein blood samples using ACCU-CHEK compact plus glucometer (Roche, France). Rats with glucose level of 300 mg/dl or higher were considered as diabetic.

### Experimental design

Normal healthy rats were used as normal control and diabetic-induced rats and were randomly divided into four groups by taking six rats in each group: (1) Vehicle treated normal rats (Cont), (2) STZ treated rats (STZ), (3) STZ + morin (15 mg/kg/day) treated rats (STZ+M15) and (4) STZ + morin (30 mg/kg/day) treated rats (STZ+M30). Morin doses were selected from literature [19,21] and the treatment was started orally by gavage after a week of STZ injection and continued for five consecutive weeks. General health and behavior of the animals were carefully monitored during the entire study. At the end of the treatment, rats were fasted overnight and under the slight anesthesia, blood samples were collected through cardiac puncture in plain sterilized centrifuge tubes. Blood samples were centrifuged at 4000 RPM for 10 minutes. Serum was suppurated and stored at −80°C till analysis. Both the femoral bones were dissected immediately and preserved for micro CT analysis.

### Assay of glucose and insulin levels

Serum glucose level were assayed using commercially available kit (RANDOX Laboratories Ltd., UK), while insulin serum level was assayed using ELISA kit (BioSource, Europe S.A., Belgium).

### Assay for bone metabolic biomarkers

Serum level of deoxypyridinoline cross links (DPD), osteocalcin (OC), bone specific alkaline phosphatase (BALP) and telopeptides of collagen type I (CTX) were determined by using ELISA kits (USCN LIFE, Wuhan EIAab Science Co., Ltd).

### Assay for inflammatory cytokines levels

Concentrations of IL-1β, IL-6, and TNF-α were determined in serum using commercially available ELISA kits (USCN LIFE, Wuhan EIAab Science Co., Ltd).

### Assessment of systemic oxidative stress

In serum, thiobarbituric acid reactive substances (TBARS) and reduced glutathione (GSH) levels were estimated considering as sensitive indicators for systemic oxidative stress. A TBARS assay kit (ZeptoMetrix Corporation, Buffalo, New York, USA) was used to measure lipid peroxidation products, malondialdehyde (MDA) equivalents. Briefly, 100 μl of serum was mixed with 2.5 ml reaction buffer (provided by the kit) and heated at 95°C for 60 min. After cooling, the supernatant’s absorbance was recorded at 532 nm. Serum GSH levels were assayed by using the method described by Sedlak and Lindsay (1968) [22]. In brief, 0.5 mL serum was mixed with 0.2 M Tris buffer, pH 8.2 and 0.1 mL of 0.01 M Ellman's reagent, [5,5'-dithiobis-(2-nitro-benzoic acid)] (DTNB). Sample tubes were then centrifuged at 3000 RPM at room temperature for 15 min. The absorbance of the clear supernatants was measured at 412 nm.

### Micro-CT analysis

Trabecular bone mineral density (BMD) and other morphometric parameters such as percent bone volume (BV/TV), structure model index (SMI), trabecular number (Tb.N), trabecular separation (Tb.Sp), trabecular thickness (Tb.Th) and trabecular porosity (Tb.Po) were measured in the right rat femoral bones head using a high-resolution, cone-Beam micro CT system (SkyScan 1172, SkyScan, Kontich, Belgium) that was kindly provided by Engineer Abdullah Bagshan Growth Factors Bone Regeneration Chair (GFBR), King Saud University, Riyadh, Saudi Arabia. In brief, bone samples were placed in a cylindrical holder, where the longitudinal axis of the bone and the sample holder were parallel to each other. Scans were done using 70 kV applied voltage with one mm aluminum filter. All cross sections contained 1024×1240 pixels with an isotropic voxel size of 10 μm. Data analysis was carried out by CT Analyzer 1.10.1.0 software (SkyScan, Kontich, Belgium). Depending on the length of the specimen, high resolution scanning was completed with slice number up to 1700. All scanning conditions and reconstruction procedure were those recommended by the manufacturer [23].

### Statistical analysis

Data were expressed as means±S.E.M. Statistical analysis was carried out using one-way ANOVA followed by Newman-Keuls as post hoc test. P value of ≤ 0.05 was considered statistically significant. All statistics tests were conducted using Graph Pad Prism (version 5) software.

## Results

### Effects on body weight, blood glucose and insulin levels

**Figure 1:**
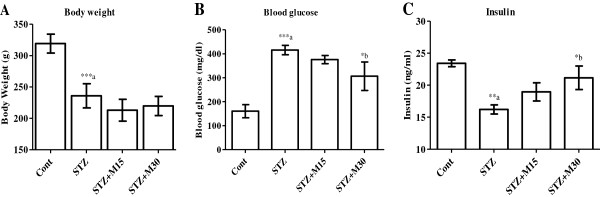
**Effects of morin on (A) animals final body weight, (B) blood glucose and (C) insulin levels.** Data were expressed as Mean±S.EM (n=6) and analyzed using one-way ANOVA followed by Student Newman-Keuls as post hoc test. ***a P<0.001 and **a P<0.01 Control vs STZ group; *b P<0.05 STZ vs. STZ+M15 or STZ+M30 groups.

Mean body weights are significantly (P<0.001) decreased in diabetic rats as compared to control animals. Morin treatments could not correct the body weights significantly (Figure 1A). Fasting blood glucose levels were significantly (P<0.001) increased and insulin levels were significantly (P<0.01) decreased in STZ group as compared to control animals. In higher dose (30 mg/kg/day) morin treated group, glucose levels were significantly (P<0.05) reduced and insulin levels were significantly (P<0.05) increased compared to untreated diabetic rats (Figure 1B & C).

### Micro-CT analysis

**Figure 2:**
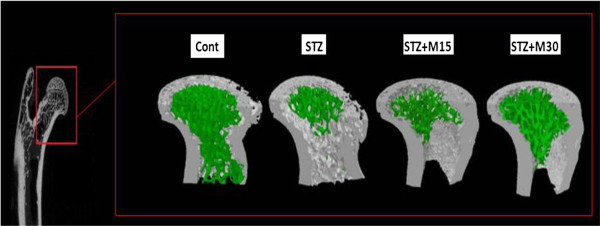
3D micro CT image showing morin protective effects on trabecular bone (Green portion) of the femur head of normal and diabetic rats.

Experimentally-induced diabetes rats resulted impairment in BMD significantly compared to control rats. In diabetic group, there was a significant decrease in BV/TV, Tb.N and Tb.Th (P<0.01, P<0.01, P<0.05 and P<0.01, respectively) and increase in SMI, Tb.Sp and Tb.Po (P<0.01, P<0.05 and P<0.05, respectively) as compared to control group of animals. Morin treatment with higher taken dose showed significant improvement in trabecular bone mass loss and microarchitecture deterioration in diabetic rats. BMD, BV/TV, Tb.N and Tb.Th values were significantly (P<0.05) higher, and the values of Tb.Sp and Tb.Po were significantly (P<0.05) lower in morin treated diabetic rats (30 mg/kg) compared to untreated diabetic rats (Table 1). As shown in the 3D micro CT image (Figure 2), the green area justified the above Table 1 parameters and showed morin protective effects on trabecular bone of the femur head of normal and diabetic rats.

**Table 1:** Effects of morin on trabecular morphometric parameters measured by micro-CT in femur head of diabetic rats

	**Control**	**STZ**	**STZ+M15**	**STZ+M30**
BMD (g/cm^3^)	0.772±0.022	0.627±0.011^**a^	0.653±0.034	0.732±0.025^*b^
BV/TV (%)	0.444±0.021	0.277±0.021^**a^	0.341±0.018	0.369±0.028^*b^
SMI	0.874±0.101	1.655±0.101^**a^	1.381±0.131	1.298±0.137
Tb.N (mm^-1^)	3.534±0.143	2.515±0.197^*a^	2.914±0.165	3.339±0.202^*b^
Tb.Sp (mm)	0.173±0.011	0.224±0.009^*a^	0.197±0.002	0.176±0.009^*b^
Tb.Th (mm)	0.123±0.002	0.107±0.002^**a^	0.115±0.001	0.118±0.002^*b^
Tb.Po (%)	58.614±1.382	66.537±0.611^*a^	61.038±1.643	58.285±2.532^*b^

Data were expressed as Mean±S.EM (n=6) and analyzed using one-way ANOVA followed by Student Newman-Keuls as post hoc test. ^*a^ P<0.05 and ^**a^ P<0.01 Control vs STZ group; ^*b^ P<0.05 STZ vs. STZ+M15 or STZ+M30 groups.

### Effects on bone metabolic biomarkers

**Figure 3:**
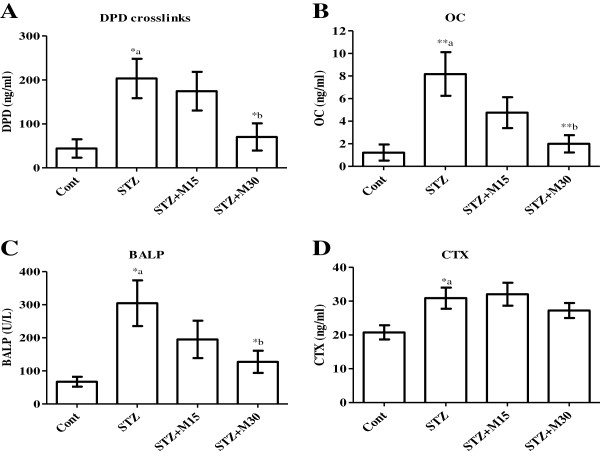
**Effects of morin on serum levels of (A) DPD, (B) OC, (C) BALP and (D) CTX in diabetic rats.** Data were expressed as Mean±S.EM (n=6) and analyzed using one-way ANOVA followed by Student Newman-Keuls as post hoc test. *a P<0.05 and **a P<0.01 Control vs STZ group; *b P<0.05 and **b P<0.01 STZ vs. STZ+M15 or STZ+M30 groups.

Serum levels of bone metabolic biomarkers including DPD (Figure 3A), OC (Figure 3B), BALP (Figure 3C) and CTX (Figure 3D) are significantly (P<0.05) increased as compared to levels in control group. Morin treatment only at higher dose (30 mg/kg/day) for 5 consecutive weeks to diabetic rats showed significant inhibition in DPD (P<0.05), OC (P<0.01) and BALP (P<0.05) biomarkers respectively.

### Effects on serum IL-1β, IL-6 and TNF-α

**Figure 4:**
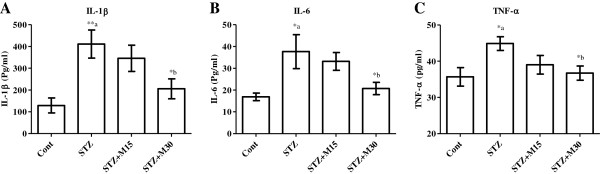
**Effects of morin on serum levels of (A) IL-1β, (B) IL-6 and (C) TNF-α in diabetic rats.** Data were expressed as Mean±S.EM (n=6) and analyzed using one-way ANOVA followed by Student Newman-Keuls as post hoc test. *a P<0.05 and **a P<0.01 Control vs STZ group; *b P<0.05 STZ vs. STZ+M15 or STZ+M30 groups.

In diabetic rats, serum IL-1β (Figure 4A), IL-6 (Figure 4B) and TNF-α (Figure 4C) levels were significantly P<0.01, P<0.05 and P<0.05 increased compared to control group respectively. Higher taken dose of morin, significantly (P<0.05) reduced the elevated interleukins and TNF-α levels in comparison to untreated diabetic rats respectively.

### Effects on systemic oxidative stress

**Figure 5:**
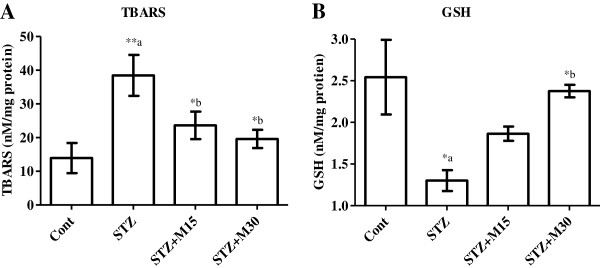
**Effects of morin on serum levels of (A) TBARS and (B) GSH in diabetic rats.** Data were expressed as Mean±S.EM (n=6) and analyzed using one-way ANOVA followed by Student Newman-Keuls as post hoc test. *a P<0.05 and **a P<0.01 Control vs STZ group; *b P<0.05 STZ vs. STZ+M15 or STZ+M30 groups.

Serum TBARS levels (Figure 5A) significantly (P<0.01) increased while GSH (Figure 5B) decreased significantly (P<0.05) in diabetic rats as compared to control animals. Morin administration following higher dose significantly (P<0.05) decreased the elevated TBARS levels and increased the reduced GSH levels as compared to STZ group.

## Discussion

Experimentally induced diabetes by streptozotocin (STZ) in rodents is a well known animal model that has been used extensively in several metabolic and pharmacological investigations [24,25]. Intraperitoneal STZ injection to the animals results in a prompt pancreatic β-cells destruction, diminish the insulin levels and induce significant hyperglycemia [24]. Similar results are found in the present study. Rats treated with morin (30 mg/kg/day) to diabetic rats for a period of 5 weeks significantly decreased the levels of glucose and increased insulin levels compared to untreated diabetic rats. There is increasing evidence of potential benefits of phenolic compounds in the cellular regulations such as redox control and inflammatory responses, and thus may protect against diabetes [26,27]. In addition, one *in vivo* study suggested that quercetin, a phenolic compound, has a protective effect in diabetes by decreasing oxidative stress and preserving pancreatic β-cell integrity, possibly through decreasing lipid peroxidation and nitric oxide production, as well as by increasing antioxidant enzyme activities [28]. All of these studies suggest that the application of phenolic compounds might positively preserve β-cells and their function. In present study, morin revealed hypoglycemic effect and also enhanced the insulin sensitivity, this may suggest that, morin has antidiabetic potentials.

Hyperglycemia is suggested to be implicated in the development of diverse diabetic complications, such as retinopathy, nephropathy, neuropathy, etc. [29]. Studies showed that the resulted hyperglycemia is associated with impairments in the micro-architecture of animal bones [25]. Thrailkill et al. reported that, hyperglycemia partially altered micro-structure of trabecular and cortical bone [30]. In several experimental studies done on different animals showed that, STZ cause impairment in serum bone biomarkers [3,31,32]. Likewise in the present study, sings of bone resorption and inhibition of formation were observed and the data revealed that the STZ injection significantly elevated the bone biomarkers named DPD, OC, BALP and CTX. In addition, micro CT analysis showed a decrease in trabecular BMD, BV/TV, Tb.N and Tb.Th and an increase in SMI, Tb.Sp and Tb.Po in diabetic rats.

Various mechanisms have been suggested to be responsible for the pathogenesis of diabetic-induced osteopenia. One of the most widely accepted mechanisms is the oxidative stress associated with hyperglycemia. Earlier studies documented that hyperglycemia can trigger oxidative stress resulting in alteration of bone metabolism and architecture [2,3,33]. Oxidative stress can also considerably provoke the cellular dysfunctions and damage in a variety of cell types such as bone cells via generating free radical known as ROS [2,4,5]. Both TBARS and GSH can be considered as sensitive parameters for oxidative stress. TBARS is a byproduct of lipid peroxidation process following free radical like ROS reaction with membrane lipids. On the other hand, glutathione is an important intrinsic non-enzymatic antioxidant. It is a free radical scavenger which maintains cellular normal redox state and neutralizes all the deleterious effects of oxidative stress [10]. Increase in serum TBARS level and decrease the GSH level, as showed in our results that an indication for elevated systemic oxidative stress. These finding are in agreement with other studies where systemic oxidative stress was found to be associated with experimentally induced diabetes [3,31]. In addition, hyperglycemia itself can potentiate oxidative stress process. High blood glucose level has been reported to trigger oxidative stress process in many cell types under diabetic conditions via both increased production of ROS and decreased endogenous antioxidant capacity [34,35]. Inflammation is another possible mechanism that might be involved in bone loss associated with diabetes. Diabetic conditions are well known to increase the level of inflammatory cytokines such as IL-1β, IL-6 and TNF-α [36,37]. Numerous studies indicated the involvement of these cytokines in the process of bone loss with or without diabetes [7,31,38,39]. The process of the development and activation of bone resorping cells, osteoclasts, was found to be strongly potentiated by these cytokines via binding to their own specific receptors on the surface of the osteoclasts [38]. Similarly in present study, IL-1β, IL-6 and TNF-α level were significantly elevated in diabetic animals. We suggest that the inflammatory conditions associated with experimentally induced diabetes have activated the process of osteoclastogenesis, which may explain the impairment in bone metabolic markers noticed in our study.

Interest in the possible health benefits of flavonoids has increased owing to their potent antioxidant and free-radical scavenging activities observed *in vivo* and *in vitro* studies. Morin is one of them and has been used in herbal medicines and for food preservatives [40]. Several biological properties have been suggested for morin, including antioxidant and/or free radical scavenger properties, in different tissues, such as the cardiovascular and hepatic tissues [41-43]. Flavonoids, in general, are now well recognized to possess numerous pharmacological activities, mainly through their ability to inhibit enzymes and/or their antioxidant properties [44] and are powerful free radical scavengers [45]. Epidemiologic studies exploring the role of flavonoids in human health for protecting with their abilities to cardiovascular disease, cancer and diabetic-induce disorders such as neuropathy, retinopathy and osteopenia. Although few studies demonstrated no effects, and a few studies suggest potential harm [46-48]. However, there are no reports on morin toxicity in any of the preclinical and clinical studies. Thus, the present pre-clinical study showing potential on diabetic-induced osteopenia will be good an opening to use morin for clinical trials. Interestingly, some of these flavonoids were found to attenuate bone loss and osteopenia associated with experimentally induced diabetes in rodents. Liang et al. reported that, quercetin can alleviate the diabetic induced impairment in BMD, micro architecture of the bone as well as biochemical and mechanical markers for bone turnover [3]. Furthermore, using dual-energy X-ray absorptiometry (DEXA), Pohaci et al. found that chronic administration of natural polyphenols can also ameliorates diabetic induced alteration in BMD [49]. Similar results were found in present study, as morin showed protective effects against diabetic induced bone loss in rats. Morin, especially at the higher dose, significantly attenuated the increased levels of bone turn over biomarkers, DPD, OC and BALP. In addition, the impaired values of trabecular BMD, microarchitecture and morphometric parameters were significantly corrected after morin administration for 5 consecutive weeks to diabetic rats. The antioxidant, anti-inflammatory and blood glucose lowering properties of morin might explain its osteo-protective effects against diabetic induced osteopenia in the current study. Morin is a potent antioxidant, which was found to exert strong inhibitory effect on ROS generation [8]. It is also an effective free radical scavenger [50]. Morin was reported to restore the expression, activities of antioxidant enzymes and levels of GSH [8]. Morin significantly attenuated oxidative stress and brought the decreased of SOD, CAT, and GPx activities and the increased levels of TBARS and hydroperoxides to near normal values in rats were having mammary carcinoma [10]. However, morin has that antioxidant property was shown to protect various human cells, such as myocytes, endothelial cells, hepatocytes and erythrocytes, against oxidative damages [17,18]. The hydroxyl groups present at the C-3 and C-5, besides at C-4 are suggested to be responsible for morin beneficial properties and is considered contributory for its antioxidant activity via quenching free radicals generated during oxidative stress conditions [51]. Both the doses of morin in present study, significantly inhibited TBARS elevated levels, while only the higher dose restored GSH reduced level in diabetic rats. In addition, morin higher dose significantly lowered the level of blood glucose after 5 weeks of treatment in diabetic rats. This may result of inhibition in systemic oxidative stress because hyperglycemia has been recognized to trigger oxidative stress and to be involved in the pathogenesis of osteopenia in association of diabetes [3,4]. The anti-inflammatory properties of morin were reported in several *in vitro* and *in vivo* studies [52-54] and it was found to have beneficial effects on inflammatory diseases such as colitis in rats [52,55]. Recently, Chen and his colleagues reported that, morin suppressed the production and expression of several inflammatory mediators including NO, PGE-2, iNOS and COX-2 [56]. Similarly, the results in present study revealed the potentials of morin (30 mg/kg) against elevation of inflammatory cytokines IL-1β, IL-6 and TNF-α in diabetic animals, which supports its anti-inflammatory property and also its possible beneficial effects in diseases where inflammation is deemed to play a pathological role.

In conclusion, our results revealed the positive effect of morin against diabetic associated impairment in bone metabolism, density and architecture in rats. Such osteo-protective effects might be explained through direct or indirect ways of its potent anti-inflammatory and antioxidant capabilities to lower bone loss involved by the diabetic conditions. Although, the osteo-protective properties of morin against diabetic induce osteopenia were identified in this study, the mechanisms underlying the effect of morin on oxidative stress and inflammation under diabetic condition still need to be identified in future studies.

## Competing interests

The authors declare that they have no competing interests.

## Authors’ contributions

MMA and MYP have performed experimental designed, induction of diabetes and animal treatment. HMA and SS Al-R have carried out biochemical and statistical analysis as well as interpretation of the data. HMA and KA Al-H shared micro CT analysis. MMA participated with HMA in histopathological investigation and writing of the manuscript. SS Al-R has revised and submitted the final manuscript. All authors read and approved the final manuscript.
